# A Low-Cost Validated Two-Camera 3D Videogrammetry System Applicable to Kinematic Analysis of Human Motion

**DOI:** 10.3390/s25164900

**Published:** 2025-08-08

**Authors:** Alejandro Peña-Trabalon, Salvador Moreno-Vegas, Maria Belen Estebanez-Campos, Fernando Nadal-Martinez, Francisco Garcia-Vacas, Maria Prado-Novoa

**Affiliations:** Clinical Biomechanics Laboratory of Andalusia (BIOCLINA), 29071 Málaga, Spain; salvador.moreno@uma.es (S.M.-V.); belen@uma.es (M.B.E.-C.); fnm@uma.es (F.N.-M.); fgv@uma.es (F.G.-V.); maria.prado@uma.es (M.P.-N.)

**Keywords:** videogrammetry, low-cost, kinematic analysis, human motion

## Abstract

**Highlights:**

This work presents a low-cost 3D videogrammetry system of high applicability. The system has been validated against Vicon^®^, a widely used commercial solution in mus-culoskeletal kinematics analysis, demonstrating comparable accuracy and repeatabil-ity. Its accessible design makes it a practical alternative for motion analysis applica-tions, with potential use in both musculoskeletal studies and general kinematic evalua-tions.

**What are the main findings?**

- It is a low-cost, high precision 3D videogrammetry system.
- It has similar accuracy and repeatability to Vicon^®^, a commercial system for kinematic analysis of musculoskeletal models.

**What is the implication of the main finding?**

- It has increased accessibility to vision systems for kinematic analysis with high accuracy for research and clinical applications.
- It has greater modularity than commercial systems.

**Abstract:**

(1) Background: Image acquisition systems based on videogrammetry principles are widely used across various research fields, particularly in mechanics, with applications ranging from civil engineering to biomechanics and kinematic analysis. This study presents the design, development, and validation of a low-cost, two-camera 3D videogrammetry system for the kinematic analysis of human motion. (2) Materials and Methods: Built using commercially available components and custom MATLAB^®^ (version 2019b) software, the system captures synchronized video streams and extracts precise 3D coordinates of markers. Its performance was validated against the Vicon^®^ (Vicon Nexus 1.7.1) system, a gold standard in musculoskeletal motion analysis. Comparative tests were conducted under static and dynamic conditions at varying working distances and velocities. (3) Results: Results demonstrate that the proposed system achieves high accuracy, with maximum measurement errors below 0.3% relative to Vicon^®^, and similar repeatability (SD of approximately 0.02 mm in static conditions). Compared to manual caliper measurements, both vision systems yielded similar results, with errors ranging between 0.01% and 0.82%. (4) Conclusions: A low-cost, two-camera videogrametric system was validated, offering full transparency, flexibility, and affordability, making it a practical alternative for both clinical and research settings in biomechanics and human movement analysis, with potential to be extended to general kinematic analysis.

## 1. Introduction

Kinematic analysis of human motion is currently predominantly conducted using image capture systems, due to their ease of implementation and the efficiency with which results can be obtained. This analysis holds significant potential in fields such as biomechanics, sports science, computer animation, motion control, and the development of virtual reality environments [1,2,3,4,5]. In this context, photogrammetry and videogrammetry represent two of the most extensively used techniques.

Photogrammetry enables the measurement of geometry, displacement, and deformation of a structure through the analysis of images [6]. With the advancement of technology and the increasing availability of low-cost, high-resolution cameras, the application of photogrammetry has expanded beyond traditional domains into fields such as mechanical and civil engineering [7,8,9,10,11,12].

On the other hand, videogrammetry involves the acquisition of video sequences using video cameras and their subsequent processing through photogrammetric algorithms [1]. This method offers several advantages, including the ability to capture a significantly larger volume of data and dynamic information, thereby enabling temporal analysis of the system under study [2]. However, one notable limitation of videogrammetry is the lower resolution of video frames. Recent technological advancements, particularly in camera hardware and data processing capabilities, are progressively overcoming this limitation by enabling higher frame rates and improved image quality.

Both techniques can be used to extract 2D and 3D information from human motion. While 2D analysis generally requires a single image acquisition device, 3D reconstruction requires the use of at least two synchronized cameras.

The advances in 3D techniques have enabled the development of systems with point tracking algorithms [13] based on the use of multiple cameras and the principles of stereovision [14,15,16], which are essential in 3D kinematics analysis. Tracking specific elements or markers over time facilitates the collection of extensive temporal data, allowing for the analysis of motion patterns, behavior, and kinematic changes across various movements and scenarios [17,18,19,20,21].

Recently, alternative approaches based on artificial intelligence have emerged [22], enabling kinematic analysis of human motion without the need to attach markers to the subject. However, such systems are limited to analyzing movement patterns for which they have been previously trained, making their applicability less general.

Commercially available systems for kinematic analysis, like Vicon^®^, Qualsys^®^, and Optitrack^®^, among others, are typically associated with high costs, limiting their accessibility for research and clinical applications. This paper introduces a low-cost, 3D videogrammetry system, detailing its design, development, and validation for use in kinematic analysis of human movements without specific constraints, with potential for extension to kinematic measurements in other domains. The system was developed within the MATLAB^®^ environment (MathWorks, Natick, MA, USA).

## 2. Materials and Methods

The components required for the proposed low-cost system are organized into the following five main subsystems: the vision system, optics, calibration pattern, markers placed on points of interest, and the lighting system.

To validate the accuracy and reliability of the proposed system, its measurements were compared against those obtained using Vicon^®^ (Vicon Nexus 1.7.1, Oxfordshire, UK), a widely recognized commercial system commonly employed in kinematic analysis of human motion.

### 2.1. Materials

#### 2.1.1. Vision System

**Figure 1:**
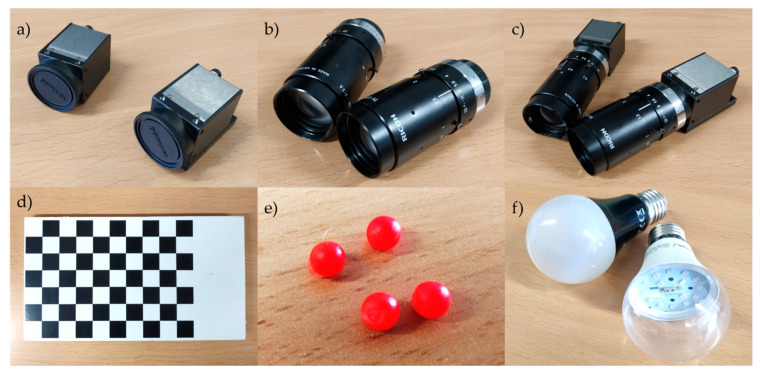
Element composing the proposed videogrammetry system: (**a**) digital camera Baumer, model VCXU-124C; (**b**) optics Pentax TV lens; (**c**) cameras with the optics; (**d**) calibration pattern; (**e**) markers coated with red reflective paint; (**f**) black light bulbs Velleman.

Implementing 3D videogrammetry requires the use of at least two cameras, which must be synchronized to ensure simultaneous image acquisition. Ideally, cameras capable of capturing color images and video sequences are preferred. Figure 1a displays the high-speed Digital Baumer camera, model VCXU-124C (Baumer, Frauenfeld, Switzerland), employed in the proposed system. This camera features a 1.1 CMOS sensor with a resolution of 4096 × 3000 pixels, a color depth of 12-bit raw (Bayer), supporting various output formats, including Mono8/10/12 and RGB8/BGR8, and operates at a frame rate of up to 29 fps (frames per second).

To maintain the integrity of the calibration and ensure accurate data collection, the cameras must remain fixed in space throughout the acquisition process. Any displacement during the experiment could compromise calibration accuracy. To avoid such issues, stable mounting solutions, such as tripods, are recommended. In this work, Manfrotto 808RC4 (Manfrotto, Cassola, Italy) tripods were used, providing high stability and allowing for precise camera positioning, with six degrees of freedom.

#### 2.1.2. Optics

The optical components used in each camera are shown in Figure 1b and correspond to Pentax TV lens models (Pentax, Tokyo, Japan). These lenses have a fixed focal length of 75 mm and a maximum aperture of f/2.8. The only adjustable parameter is the focus distance (depth of field), which ranges from 0.5 m to infinity. The complete vision system setup, including the mounted optics, is illustrated in Figure 1c.

#### 2.1.3. Calibration Pattern

A predefined calibration pattern is needed for the spatial synchronization of the vision system and for determining the intrinsic and extrinsic parameters of each camera. The checkerboard size should enable capturing sufficient calibration images within the smallest working volume; in our case, it was chosen to fit approximately 8 times within the image plane for the smallest working volume when the pattern was placed parallel to the image plane and roughly at the center of the workspace. The calibration pattern shown in Figure 1d was specifically designed for the proposed videogrammetry system. It consists of a 10 × 7 grid of black and white squares, where the control points for calibration are the 6 × 8 internal corners of the checkerboard, as corners are extremely small and often invariant to perspective and lens distortion. The calibration pattern must be printed on a flat, matte surface to minimize reflections that could interfere with accurate feature detection. For the validation of the system, it was printed on a PVC board of 5 mm thickness to ensure planarity. The size of the printed checkerboard squares was measured by 10 subjects, each performing 10 measurements using a caliper (Mitutoyo, Model 500-196-30, Kawasaki, Japan), resulting in 13.53 ± 0.01 mm.

#### 2.1.4. Markers

The identification of points of interest is a critical step in this videogrammetry system, as it directly impacts the accuracy of the 3D data used for kinematic analysis. Spherical markers must be used, since the system is based on the computation of the position of the markers’ centroid in the image plane, and only spherical surfaces maintain the centroid in the same position when projected onto any spatial plane.

Although no specific optical characteristics are required for the marker surfaces, it is recommended to use markers (Figure 1e) that reflect a particular wavelength band that is detectable by the video cameras, preferably non-visible, such as ultraviolet light (UV-A), in combination with a lighting system emitting in the same wavelength band (Figure 1f).

This approach facilitates marker tracking by the software and improves the user-friendliness of the system. In cases where the system being measured exhibits significant color variation, reflective markers may not be necessary. Instead, markers that provide adequate visual contrast with the rest of the image, such as those of a very different color, may be sufficient. However, gray markers are not recommended under visible light, as they can be confused with their own shadows by the tracking system.

#### 2.1.5. Lighting System

To ensure optimal marker visibility, illumination must be homogeneous and uniformly distributed across the scene, effectively lighting the markers from all directions and minimizing shadow formation.

A highly effective system involves the use of reflective markers. In such cases, if visible light is attenuated during acquisition and illumination is provided exclusively by a source emitting within the specific wavelength band reflected by the marker surfaces, the contrast of the markers is significantly enhanced relative to the rest of the scene. As a result, the corresponding marker pixels become highly prominent and are almost exclusively visible in the image planes.

### 2.2. Methods

#### 2.2.1. Videogrammetric Process

**Figure 2:**
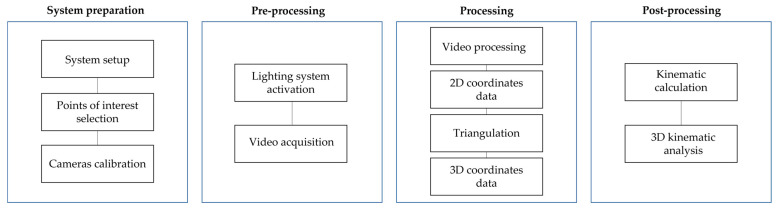
Videogrammetry system workflow.

The complete workflow for obtaining 3D coordinates and performing kinematic analysis is illustrated in Figure 2.

The process begins with the assembly of all components of the videogrammetry system, including mounting the cameras with their respective optics onto tripods and proper positioning of the cameras. Cameras must maximize marker coverage throughout the test, i.e., be positioned to keep all markers within the image planes for the duration of the entire measurement process with minimal occlusion and overlap. If a marker is occluded or overlapped in a given frame, its position cannot be calculated and must be estimated from neighboring frames. Cameras and tripods must remain stable and unchanged throughout testing. The lighting system must be illuminated all markers uniformly from all directions, without any part of the surface appearing in shadow at any time during the trial.

##### Calibration

At least two synchronized cameras are required for 3D data acquisition. This synchronization must be both temporal and spatial. In the proposed system, temporal synchronization was achieved using an external trigger, which delivered a simultaneous signal to both cameras.

For spatial calibration, a stereo calibration procedure is performed after camera positioning to determine intrinsic and extrinsic parameters of each camera, i.e., the internal characteristics of each camera (such as focal length and lens distortion) and the relative spatial positioning between the cameras, respectively.

**Figure 3:**
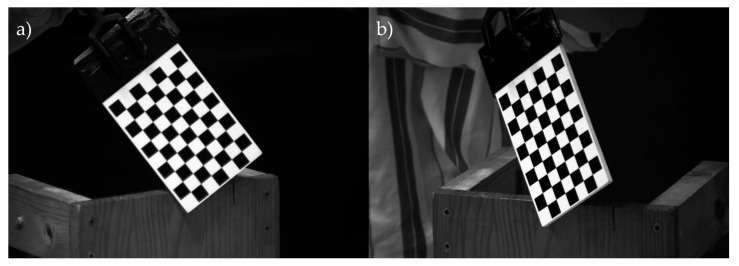
Images of the calibration pattern taken during the calibration process of the videogrammetry system by the (**a**) camera placed in the left side; (**b**) camera placed in the right side.

There are between 10 and 30 image pairs of the calibration pattern of Figure 1d, ensuring the full pattern is fully visible in each image and captured simultaneously by both cameras (Figure 3). These images must cover the entire working volume, while maintaining a maximum inclination of 45° to avoid distortions.

Calibration images are processed using the Stereo Camera Calibrator toolbox in Matlab^®^ (MathWorks, Natick, MA, USA) by specifying the known square size in the pattern (13.53 mm in this study). The toolbox outputs the intrinsic and extrinsic camera parameters and calibration error.

These parameters are subsequently used for triangulation, converting 2D image data into accurate 3D spatial coordinates.

##### Processing Software

The software (provided as Supplementary Material) processes the recorded videos. Each video is initially decomposed into frames, and each frame is sequentially analyzed.

Upon launching the application, the video files are selected. First, the user is prompted to specify the number of markers; select the marker color from red, green, blue, black, or white; and provide an estimate of the initial value for the radii of the markers. To define these radii, the user is required to click on two diametrically opposite points on the edge of any marker in the initial frame of each camera. The radii will be used to initiate the marker identification in each camera and to define the radius of the structuring element for morphological closing (explained below). The user is then prompted to select a pixel belonging to any of the markers to be analyzed in a grayscale image of the first frame. Preferably, this pixel should be chosen close to the edge of the marker, as edge pixels typically have lower grayscale values than the central areas of the markers, despite uniform and homogeneous lighting of the scene. This behavior was consistently observed in the numerous tests we conducted during the development of the algorithm. This recommendation is explicitly presented to the user during the pixel selection step. Next, the user is prompted to decide the order for the marker labeling. To define this order, the user is asked to select a pixel approximately at the center of each marker in the first frame of the recording following the intended sequence. Consistent marker ordering between both video streams is essential to address the data association problem.

Color-based filtering is applied in the HSV (Hue, Saturation, Value) color space, based on the hue specified for the markers. This color filtering retains only areas that match the target color in each frame. Eight-bit binarization is then applied to each frame, assigning 0 to non-retained pixels and values from 1 to 255, based on their intensity, to the other ones. A minimum area filter of 50 pixels^2^ (user-adjustable) removes irrelevant small isolated regions unrelated to the markers. Next, morphological closing, using a disk-shaped structuring element with a radius set to 20% (user-adjustable) of the marker diameter previously selected by the user, smooths region boundaries and fills any holes. The radius is not a fixed value but is dynamically adapted according to the specific characteristics of each test and the size of the markers.

Finally, to refine detection accuracy, a grayscale intensity threshold is applied, discarding regions with insufficient color intensity that may otherwise produce false positives. When working with markers of colors other than black, the code operates with a threshold set at 20% (user-adjustable) below the gray level of the pixel selected by the user in the initial frame. Therefore, any pixels with grayscale levels below the threshold are set to 0, while the other ones are assigned a value of 255, forming the region of interest. When working with black markers, the threshold is set to a value 20% (user-adjustable) higher than the grayscale level of the selected pixel within the marker. In these cases, the objective is to eliminate the pixels that constitute the markers themselves. To accomplish the task, a complementary image is generated, in which the pixels corresponding to the removed markers are assigned a value of 255 to form the regions of interest.

If the algorithm fails to accurately identify markers using the default parameters in a particular test, the parameters previously indicated as user-adjustable can be manually readjusted.

Marker identification starts for each camera in the first frame, as described above. The coordinates of each marker in the 2D camera plane reference system are computed as the center of the previously identified region of interest. The markers are then tracked in the subsequent frames by following the process previously described for the first frame but restricted to a 10 × 10 pixel area (user-adjustable) around the positions of each marker in the previous frame. That is, the coordinates detected in each frame serve as a reference for the next, streamlining the identification process and reducing computational time. This iterative procedure is applied across all captured frames.

A trajectory validation mechanism is integrated to recover from occasional tracking failures. If the algorithm fails to find a marker within the expected search region (i.e., resulting in a black pixel output), the user is prompted to re-identify the marker. A red indicator shows which marker requires re-identification, after which, automatic tracking resumes.

Once all frames are processed, the 2D marker trajectory of each marker in each camera plane is generated. Using the intrinsic and extrinsic calibration parameters, lens distortion correction and triangulation are performed to obtain the 3D coordinates of each marker, expressed in millimeters by default. The resulting 3D coordinate vectors are saved in both .mat (MATLAB^®^ format) and .txt file formats for further analysis.

In the post-processing, various kinematic parameters relevant to human movement can be analyzed, such as body segment lengths, as well as their velocities and accelerations. Segment lengths can be calculated using Euclidean distance between marker coordinates if they are positioned for this purpose. Velocities can be computed by deriving marker displacement over time, and accelerations from velocity changes.

Another key parameter in human movement analysis is the angle between adjacent segments, which can be computed using rigid body representations of each segment, although the calculation strongly depends on the specific model used for the musculoskeletal segment being studied.

#### 2.2.2. System Performance Evaluation

To evaluate the accuracy of the proposed videogrammetry system, the commercial system Vicon^®^ was selected as a reference for comparative validation, due to its widespread use in analyzing kinematics of musculoskeletal models. The cameras used by the Vicon^®^ system were MX-T010. These cameras feature a CMOS sensor with a resolution of 1120 × 896 pixels, a color depth of 10-bit grayscale, and can operate at a frame rate of up to 250 fps at full-frame resolution. The cameras were equipped with Pentax TV lenses (Pentax, Tokyo, Japan) with a focal length of 8.5 mm and a maximum aperture of f/1.5. Tests were conducted to compute the relative 3D coordinates of three markers, which were simultaneously recorded using both the Vicon^®^ system with just two cameras and the proposed system. This approach allows for a direct comparison of the reliability of both systems and for assessing whether the performance of the new system differs from the Vicon^®^ system.

**Figure 4:**
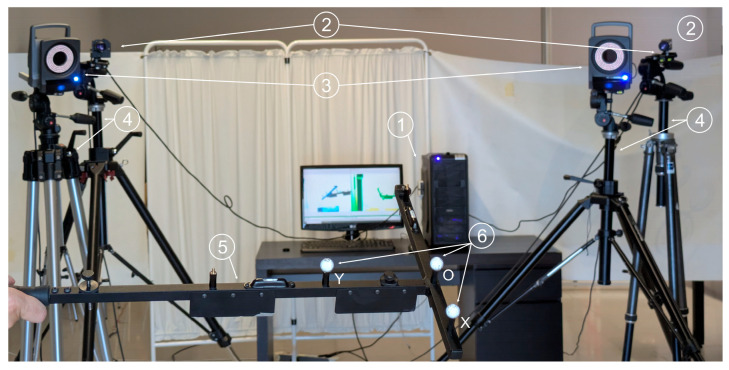
Experimental setup showing computer used to process the captured images (1); cameras used in the proposed videogrammetry system (2); cameras used in the Vicon system (3); tripods (4); rigid support for the markers (5); and markers (6).

Three Vicon^®^ markers of 14 mm diameter (reported by the manufacturer) and gray color were mounted in a matte black rigid support to ensure that their relative positions remained constant. The markers are coated with a material reflective to infrared light, enabling their detection by the Vicon^®^ system. For the proposed videogrammetry system, a non-visible wavelength band, as recommended in Section 2.1.5, was not used. Instead, only natural and ambient visible light was employed in order to avoid interfering with the infrared illumination required by the Vicon^®^ system, and because sufficient visual contrast between the markers and the support structure was anticipated. Despite the considerations discussed in Section 2.1.4, gray markers were used, since this is the visible color of the infrared-reflective coating required by the Vicon^®^ system. To minimize potential errors, a matte dark background was selected in the working area, and special care was taken to ensure that uniform lighting did not cast shadows in any direction. The arrangement of the elements of both videogrammetry systems can be seen in Figure 4.

The two cameras of each system were positioned 2.40 m apart, and each MX-T010 camera used by Vicon^®^ was placed as close as possible to the corresponding Baumer^®^ (Baumer, Frauenfeld, Switzerland) camera used in the proposed system. This configuration aimed to minimize any potential bias due to differences in camera positions relative to the markers.

**Figure 5:**
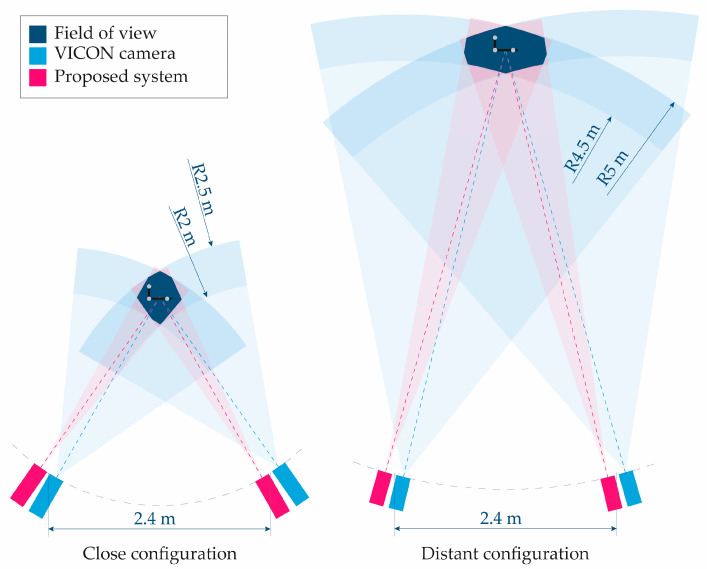
Scheme of the experimental setup showing the cameras of both the VICON system and the proposed videogrammetry system and the field of view in both configurations. All dimensions are to scale, except for the support structure with the markers.

Three tests were performed within a close-range working volume, specifically ensuring that the markers moved with a distance between 2.00 and 2.50 m inside the corresponding field of view of the four cameras. Another three tests were conducted under similar conditions but at a more distant working volume, specifically with the markers moving within a volume located at a distance between 4.50 and 5.00 m from the four cameras and within their fields of view. To orient the cameras in each configuration, a marker was placed at the center of the working space, i.e., at a distance from the four cameras equal to the average of the minimum and maximum distances that bound the working space (2.25 m for the close-range and 4.75 for the distant one), and the four cameras were then oriented so that the marker appeared at the center of all image planes. The experimental setup is depicted in Figure 5.

Once the cameras were positioned, the following three tests were conducted by varying the motion velocities for each marker-to-camera distance, while both systems recorded simultaneously:

- In the first test, the markers were recorded while holding static for 10 s.
- In the second test, the marker support structure was manually moved randomly at low velocity within the working volume.
- In the third test, the structure was moved similarly but at a higher velocity.

The distances between the markers O-X and O-Y in Figure 4 were computed throughout the trajectories by both systems. The mean values and standard deviations (SD) of these distances were calculated to assess system reliability. The SD represents the variability of the measurements, and the lower the value, the higher the precision of the system. The percentage errors between the mean distances obtained with the proposed videogrammetry system and those from the Vicon^®^ system were determined to quantify how far our system deviates from the values computed by the Vicon^®^, which has been regularly employed in biomechanical tests.

The relative technical error of measurement (rTEM) has been adopted by the International Society for the Advancement of Kinanthropometry (ISAK) to determine the margin of inter-systems error in anthropometric measurements [23]. It can be computed when the systems measure a specific distance, as follows:

$$rTEM_{inter}=\frac{1}{VAV}\sqrt{\frac{\sum {\left(d_{V}-d_{ps}\right)}^{2}}{2N}}$$

where $d_{V}$ is the distance measured by the Vicon^®^ system and $d_{ps}$ is the distance measured by the videogrammetry system proposed in this paper in each frame of the tests, N is the number of frames involved in all the tests, and VAV is the total average of the distance from all frames and of both systems. The $rTEM_{inter}$. values for the O-X and O-Y measurements were computed based on the four tests performed with simultaneous recording by both systems.

Additionally, the distance between the markers was measured with a digital caliper (Mitutoyo, Model 500-196-30, Kawasaki, Japan). Ten measurements were taken by ten different subjects.

#### 2.2.3. System Applicability Assessment

**Figure 6:**
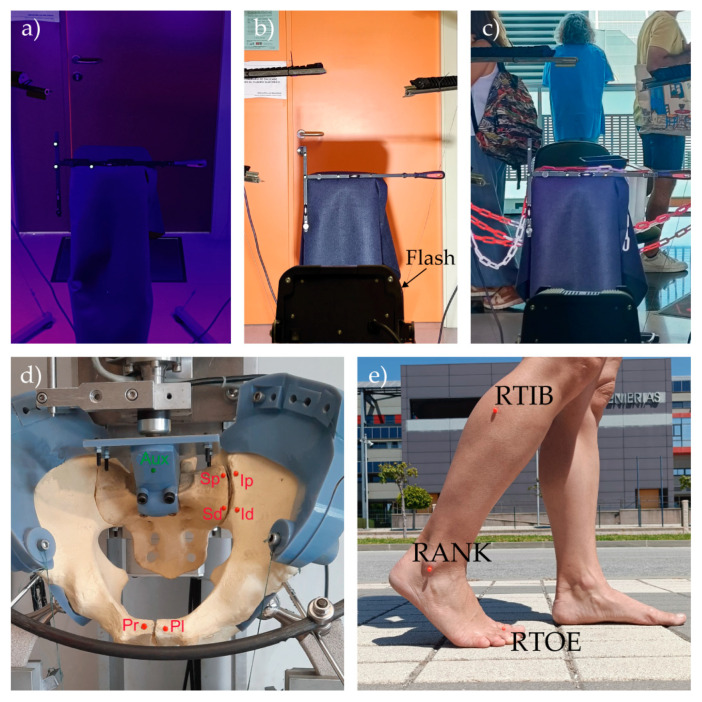
Representative images to evaluate the system’s performance (upper row) and applicability (lower row): (**a**) limited illumination; (**b**) variable illumination; (**c**) cluttered environment; (**d**) in vitro test of a fracture pelvis fixation; (**e**) in vivo gait test.

The applicability of the proposed system was further evaluated by assessing its performance under the following five different conditions: three laboratory scenarios with varying environmental conditions, one in vitro laboratory test, and one out-of-laboratory in vivo test (Figure 6). The last two tests were included solely to illustrate the versatility and applicability of the proposed videogrammetry system, rather than to perform a detailed assessment of a fracture fixation device or subject mobility.

##### Varying Environmental Conditions

To assess the proposed videogrammetry system’s performance under different environmental conditions, the marker configuration described in Section 2.2.2 was used, since the distances between markers O-X and O-Y had already been calculated and validated versus the Vicon system. As the reference system data were not required for these tests, new markers were placed at the same position with the following two objectives: on one hand, to take full advantage of the system’s capabilities, as justified in Section 2.1.4, the Vicon markers were replaced with UV-A reflective markers; on the other hand, to test the robustness of the system with different marker size and color, yellow markers of 8 mm in diameter were selected. The scenes were illuminated using four UV-A light bars (wavelength 385–400 nm) of 42w each (Onforu model CT28; Onforu, CA, USA), with two bars mounted on each vertical support, which allowed the bar light to be oriented both around the vertical pole and around its own longitudinal axis. This setup made it possible to design the illumination of the scenes, aiming to achieve the conditions recommended in Section 2.1.5, i.e., to light the markers from all directions and minimize shadow formation.

The two Baumer cameras were placed as described in Section 2.2.2 for the close configuration. The scenes were recorded for 60 s while the marker support structure was manually moved in a random manner at low-speed inside the working volume.

Three environmental conditions were reproduced, as follows:

(a) Natural light was blocked and ambient light excluded, so that the scene was illuminated exclusively by the lighting system integrated into the videogrammetry setup, in order to assess the system’s performance in extremely low-light environments, (Figure 6a).
(b) To assess the system’s performance under highly variable visible light conditions, natural light was permitted in the scene, and ambient illumination was added by orienting a white-light flash (wavelength 600 nm, 5500 K, 1000 w, positioned 1.5 m from the center of the working volume), operating at a frequency of 1 Hz, toward the center of the working area (Figure 6b).
(c) The system was evaluated in a cluttered environment. For this purpose, numerous visual stimuli (objects with surfaces of varied colors, intensities, and textures) and low contrast background elements were placed around the working volume, and the appearance of walking subjects within the image planes was planned during the recording. In no case was marker occlusion allowed. Natural light was allowed into the scene while maintaining the UV-A system illumination setup (Figure 6c).

In all three experimental conditions, the distance between the O-X and O-Y markers was calculated. The rTEM for each distance was computed as indicator of the intra-system reliability of the proposed videogrammetry system as follows:

$$rTEM_{intra}=\frac{1}{\bar{d}}\sqrt{\frac{\sum {\left(d_{i}-\bar{d}\right)}^{2}}{2N}}$$

where $d_{i}$ is the distance measured in frame i, $\bar{d}$ is the average value of the distance across all frames involved in the test, and N. is the number of frames. The $rTEM_{intra}$ was computed for the O-X and O-Y distances under the three environmental conditions studied.

##### In Vitro Applicability

To evaluate the applicability of the system, an in vitro study was conducted on a synthetic pelvic model aimed at measuring relative displacements at the pelvic joints following disruption of the left sacroiliac joint and the pubic symphysis simulating a vertically unstable pelvic injury (Tile type C1), stabilized using an external fixator [24]. Six reflective markers with a diameter of 6 mm and red color were placed on both sides of the pubic symphysis and the left sacroiliac joint. An additional single 6 mm green marker was placed at an arbitrary point on the fixture used to load the specimen (Figure 6d). The pelvic model was placed in a uniaxial testing machine [25] and subjected to a compressive load applied to the center of the L5 cranial surface from 5 N up to pelvis failure at 0.5 mm/s. A trigger signal was sent from the testing machine to the videogrammetry system to synchronize the video recordings with the pelvic loading.

The two Baumer cameras were placed 2 m apart and at a distance of 3 m from the center of the pelvic model, and they were oriented so that the center of the pelvic model appeared at the center of their image planes at the beginning of the test. Finally, the UV-A illumination system was positioned according to the recommendation in Section 2.1.5.

The videogrammetry system recorded the displacement of the seven markers during the test. The displacements at the joint were approximated by the relative displacements between markers Sp−Ip (representing the proximal displacement of the left disrupted sacroiliac joint), Sd−Id (representing the distal displacement of the left disrupted sacroiliac joint), and Pr− Pl (representing the displacement at pubic symphysis) in Figure 6d, with the displacement between markers i and j computed as follows:

$$\Delta_{i-j}=\|{\vec{X}}_{i}-{\vec{X}}_{j}\|$$

where ${\vec{X}}_{i}$ are the 3D coordinates computed for the marker i.

##### In Vivo Applicability

Marker-based models for analyzing in vivo movement of musculoskeletal segments rely on markers placed on specific anatomical landmarks and use motion capture systems to compute the 3D trajectories of these anatomical points [26,27,28].

To demonstrate the applicability of the system in assessing the human motion, the 3D coordinates of three markers placed on the right lower limb of a subject were recorded. The markers were placed on the dorsal surface of the foot over the second metatarsal head (RTOE), the lateral malleolus (RANK), and the lateral mid-shank (RTIB), corresponding with the homonymous markers of the well-known Plug-in Gait model [29]. The subject was recording while walking at her normal cadence. The test was carried out in the street to verify the performance of the system outside the laboratory. The cameras were positioned 2.5 m apart and oriented so a marker located 3 m from both cameras appeared at the center of their image plane. The subject walked on a plane approximately parallel to the camera planes, passing through the point where the marker used for alignment had been placed (Figure 6e).

## 3. Results

The distances measured with the caliper between the markers used to evaluate the system accuracy and repeatability were 79.22 (0.46) mm between O and X, and 120.27 (0.44) mm between O and Y.

### 3.1. Systems Performance Evaluation

After processing the tests, the results are based on the analysis of a total of 4400 frames. The mean marker velocity in the low-velocity tests was 9.91 ± 8.29 mm/s; in the high-velocity tests, it was 21.26 ± 12.86 mm/s.

**Figure 7:**
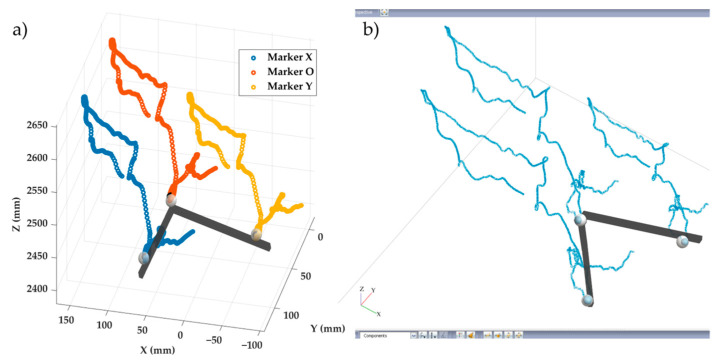
Section of the trajectories of the three markers during the close configuration test performed at higher velocity computed by (**a**) the proposed system; (**b**) Vicon^®^.

Figure 7 shows a section of the trajectories of the three markers computed by Vicon^®^ and the proposed system during the close configuration test performed at higher velocity. Table 1 shows the computed mean distances and SD between markers O-X and O-Y across the entire trajectories obtained with both systems for the six tests.

Table 2 presents a comparison of the results from both systems, detailing the percentage difference between the mean distances obtained by the proposed videogrammetry system and those obtained by Vicon^®^, as reported in Table 1 with the Vicon^®^ values used as a reference.

The $rTEM_{inter}\;$ computed to compare inter-system variability resulted in 0.22% for the distance O-X and 0.25% for the distance O-Y. The ISAK consider values below 1.5% as an excellent agreement between anthropometric measurement systems [30].

Table 3 presents a comparison of the results from the videogrammetry system and Vicon^®^ with respect to the caliper measures, detailing the percentage difference between the mean distances obtained by both systems and those obtained by the caliper.

### 3.2. System Applicability Assessment

#### 3.2.1. Varying Environmental Conditions

Table 4 presents the mean and SD of the measured distances between the O-X and O-Y markers in the three scenarios with varying lighting conditions and complexity (limited visible illumination, variable visible light conditions, and cluttered scenario), as well as the intra-system $rTEM_{intra}$ computed for each test.

$rTEM_{intra}$ values below 1% are considered to represent excellent measurement quality [30], a criterion the proposed system consistently met with values considerably lower across all trials.

#### 3.2.2. In Vitro Applicability

The experimental setup was not designed to assess the pelvic fixation system, but rather to evaluate the applicability of the videogrammetry system. Therefore, the results can only be interpreted from this latter perspective. A proper analysis of the pelvic fixation would require a detailed evaluation of marker placement and, possibly, their positioning, in order to not only study absolute joint opening, but also to analyze the motion of the bony elements involved in the model.

**Figure 8:**
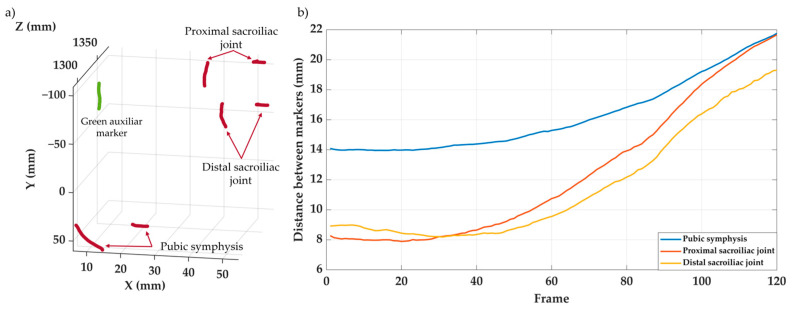
Results of the in vitro tests of the pelvic fixation system. (**a**) 3D displacement trajectories of the seven markers tracked during the test. (**b**) Evolution of joint opening at the left sacroiliac joint and the pubic symphysis.

Figure 8a shows the trajectories of the seven markers tracked during the loading test, while Figure 8b displays the evolution of the distances between the pairs of markers placed on both sides of the monitoring pelvic joint, showing that the videogrammetry system clearly captures the opening of the joints under the load. It can be observed that all seven markers were accurately tracked throughout the test, without displaying any anomalous behavior. This was consistent both in cases of small and large displacements, and regardless of the marker color. The system enabled adequate tracking of the joint opening at the sacroiliac and pubic symphysis joints, within the limitations argued above.

#### 3.2.3. In Vivo Applicability

This test was carried out just to evaluate the applicability of the videogrammetry system, as indicated in Section 3.2.2.

**Figure 9:**
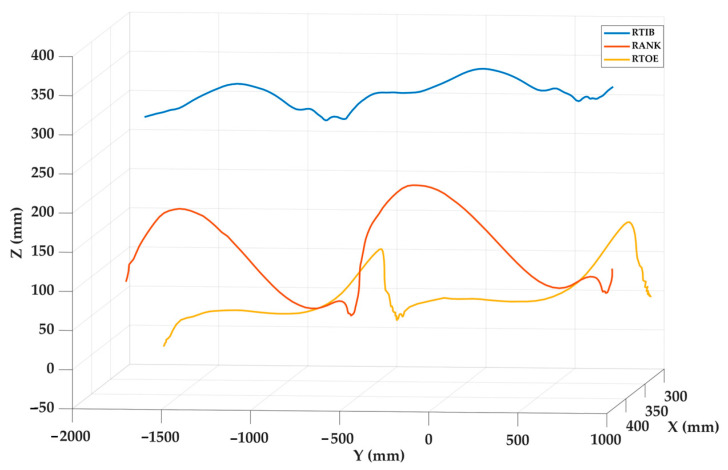
Results of the in vivo test showing the trajectories of the three markers during two steps.

Figure 9 shows the spatial trajectories of three markers during two steps. The toe and ankle markers exhibit marked vertical oscillations, with two peaks corresponding to the swing phases of two gait cycles, while the tibia follows a more horizontal path, reflecting its limited vertical displacement. This behavior is consistent with the expected biomechanics of the lower limb segments during gait. The recorded motion closely resembles normal gait patterns, highlighting the successful applicability of this vision-based system in the field of biomechanics.

## 4. Discussion

The main contribution of this work is the successful development and validation of a low-cost, non-commercial videogrammetry system. The developed system enables acquisition of 3D coordinates using two synchronized cameras, with a precision similar to the commercial software.

The robustness of the proposed algorithm was demonstrated through a comprehensive set of validation procedures under diverse experimental conditions and using markers of different sizes and colors. The system maintained high accuracy and repeatability when compared to Vicon^®^, a commercial system widely used for musculoskeletal movement analysis in both in vivo and in vitro tests [31,32,33,34], under different marker velocities and working volumes. Furthermore, it performed consistently in challenging environments, including low lighting, variable illumination, and cluttered backgrounds. Its applicability was also confirmed in both in vitro and in vivo scenarios, where the algorithm accurately captured motion data outside of controlled laboratory settings. These results support the system’s adaptability and reliability for a wide range of real-world applications.

The repeatability of the measurements taken manually with the digital caliper was much lower than those obtained with the videogrammetry systems. As shown in Table 1, the repeatability of both videogrammetry systems is very similar. In the static tests, both systems exhibited an SD of approximately 0.02 mm.

Despite the fact that the proposed system uses a purely static calibration, whereas Vicon employs a dynamic calibration, the repeatability observed during tests with moving markers is comparable. The proposed system showed SD values ranging from 0.02 to 0.25 mm, while Vicon^®^ produced SD values between 0.02 and 0.31 mm.

In terms of accuracy, considering the Vicon system as the gold standard for motion measurement in musculoskeletal systems, the errors of the proposed system relative to Vicon are very low, below 0.29%, regardless of the working volume or the marker speed within the tested ranges. With respect to manual measurements, both vision systems yielded similar results, with errors ranging between 0.01% and 0.82%. The computation of $rTEM_{inter}$ to assess inter-system variability reveals excellent agreement between the two videogrammetry systems, with values below 0.25%.

The comparative tests between the proposed videogrammetry system and the Vicon reference system were conducted under visible light conditions in order to minimize potential interference with the reference system. However, the applicability of the proposed videogrammetry system was evaluated in a variety of experiments using UV-A reflective markers and the corresponding illumination setup, as this is the configuration for which the system was designed, and which enables it to maximize its performance. Applicability tests conducted under altered visible light conditions confirmed that the system maintained its performance in the absence of ambient or natural lighting, and even under highly perturbed visible light patterns as intense flashes during recording. Similarly, it was verified that the presence of numerous visual stimuli in the scene did not reduce the effectiveness of the system, provided that the markers are not occluded. As discussed below, resolving this possible occlusion of markers would require the use of a greater number of cameras. In the three scenarios, $rTEM_{intra}$ values were below 0.11%, indicating a high degree of intra-system reliability. The experiments employed markers of different sizes (14, 8, and 6 mm in diameter) and colors (gray, yellow, red, and green), and no influence on the results was observed. Nonetheless, the authors recommend using the smallest marker size that can be tracked by the cameras, since it is known that deviations from sphericity due to manufacturing tolerances can cause shifts in the position of the marker’s centroid between different image planes, resulting in measurement errors that increase with marker size. These types of errors are inherent to any marker-based optical tracking system.

In addition, the applicability of the videogrammetry system was verified both in in vitro trials in the laboratory and for human motion tracking outside the laboratory environment. However, further testing is needed to more robustly confirm the full applicability of the system in complex real-world environments. These tests were conducted with seven and three markers, respectively. There is no reason why increasing the number of markers should compromise the accuracy or repeatability of the system, as long as no occlusion or overlap occurs between them. Under these conditions, the system’s ability to correctly identify and track each marker remains unaffected by the total number present. For this reason, and in order to isolate the core performance of the system from potential occlusion-related errors, all experiments conducted to evaluate accuracy and repeatability were performed using only three markers. The inclusion of a larger number of markers inevitably increases the computational load and slows down the image processing, but this is not considered a key limitation, since the system is specifically designed for offline use, where real-time performance is not required.

Beyond precision and repeatability, this system offers the following key advantages over commercial alternatives such as Vicon^®^: it is significantly more affordable, customizable to specific experimental needs, and fully transparent, providing access to all development processes, including software code, thereby facilitating future modifications and improvements.

Although the system with two cameras proposed in this work has demonstrated its suitability to compute the 3D coordinates of the markers with adequate accuracy and repeatability for a wide range of scenarios, two cameras may be insufficient for tracking certain activities in which markers become occluded or overlapped in the only two image planes. This issue is likely to appear when analyzing in vivo complex human movements or in in vitro experiments on articular joints or body segments.

Incorporating more than two cameras prevents the use of Matlab’s Stereo Camera Calibrator Toolbox and requires the development of a more complex calibration algorithm. If the calibration process is to be implemented in the Matlab environment, firstly, each camera must be calibrated individually to compute its intrinsic parameters (for which the *estimateCameraParameters* function is available). Secondly, one of the cameras must be selected as the reference or master camera, and the extrinsic parameters of the remaining cameras must be computed relative to it. The function proposed in this paper for estimating the extrinsic parameters between two cameras can be used for this step, as the strategy consists of building *N* − 1 stereo calibration pairs for an N-camera system, with the reference camera included in every pair. Once all intrinsic and extrinsic parameters of the calibration have been obtained, a multi-view triangulation algorithm must be run using the data captured by the set of cameras. Several strategies can be used for this purpose, such as the following: pairwise triangulation, which averages the 3D coordinates computed by all camera pairs; direct linear triangulation, an algebraic method that solves the homogeneous equation $A\cdot \vec{x}=0$, where $\vec{x}$ is the 3D coordinates of the marker and A is a coefficient matrix constructed from the 2D image projections; minimization of reprojection error, a nonlinear approach that estimates the 3D position of the marker by minimizing the total reprojection error across all views; or robust algebraic triangulation, which combines linear triangulation with techniques to compensate for noise and outliers.

Small movements of any of the cameras, which can be caused by small external perturbations, can significantly affect the calibration of a videogrammetry or photogrammetry systems due to alteration of the extrinsic parameters computed for the camera setup prior to the perturbation. This issue, common to all camera-based measurement systems, becomes particularly critical in low-cost configurations with only two cameras. In such systems, the perturbed camera will always contribute to the triangulation of marker coordinates with equal or greater weight than in systems employing a larger number of cameras. For this reason, the authors recommend performing a verification of calibration integrity prior to each new experimental trial or whenever there is any suspicion that a camera may have experienced a shift. This verification consists of recording and processing the continuous movement of a single marker, or a small group of markers, at a low velocity for a few seconds. This approach aims to ensure a low computational cost and minimal time investment in the verification step. If a perturbation has affected the system’s calibration, the reconstructed trajectory of the marker(s) will display vibratory artifacts that do not correspond to the actual movement performed, and such discrepancies would clearly indicate the need for recalibration.

Recent developments in videogrammetry have incorporated artificial intelligence (AI) techniques for the kinematic analysis of various systems. While these AI-based approaches offer significant potential [22,35], they require prior training on specific datasets to recognize the anatomical structures or motion patterns under investigation. This prerequisite limits their applicability in scenarios where such prior information is unavailable or insufficient. In contrast, the videogrammetry system proposed in this study operates independently of pre-trained models, enabling its application to complex and atypical motion analysis tasks. This feature is particularly advantageous in the study of musculoskeletal kinematics, involving uncommon movement patterns or individuals with pathological conditions that deviate from normal motion behavior. It is also worth highlighting that recent AI-based approaches using video recordings to detect human functional events, such as fall detection [36], have shown promising potential for applications in real-world scenarios.

As previously discussed, future developments will focus on extending the calibration system to allow for operation with more than two cameras, with no upper limit on the number of cameras. This upgrade would enable the execution of videogrammetric measurements in more complex scenarios, where using only two cameras may be insufficient due to the lack of two viewpoints that can guarantee proper visibility of all markers. Implementing a redundant multi-view configuration is expected to increase the system’s applicability in high-complexity contexts. Another line of future research will consist of adapting the system for use in assistive mobility structures used by individuals with motor impairments and evaluating its performance in such devices. In particular, the system is intended to be integrated as an additional sensor into a pediatric walker previously developed by the research group [37], with the objective of monitoring patients’ activity in daily-life environments that are distinct from clinical, hospital, or laboratory settings. This integration would enable the collection of motion data under real-life conditions, providing clinically meaningful insights into patients’ functional behavior during everyday activities [38].

## 5. Conclusions

A low-cost 3D videogrammetry system has been developed and validated in comparison to a commercial software, demonstrating precision and repeatability that are suitable for kinematic analysis in a wide range of scenarios.

## Figures and Tables

**Table 1:** Computed mean distances and standard deviations (mm) between O-X and O-Y using the videogrammetry system and Vicon^®^ along the trajectory for the six tests.

			Videogrammetry		Vicon^®^	
			Mean	SD	Mean	SD
Close-range volume	Statics	Distance O-X	79.69	0.02	79.61	0.02
		Distance O-Y	120.76	0.02	120.71	0.02
	Low velocity	Distance O-X	79.63	0.06	79.72	0.09
		Distance O-Y	120.48	0.16	120.66	0.06
	Higher velocity	Distance O-X	79.66	0.09	79.66	0.08
		Distance O-Y	120.28	0.14	120.29	0.05
More distant volume	Statics	Distance O-X	79.88	0.04	79.81	0.04
		Distance O-Y	120.73	0.04	120.37	0.03
	Low velocity	Distance O-X	79.72	0.19	79.76	0.23
		Distance O-Y	120.67	0.18	120.55	0.18
	Higher velocity	Distance O-X	79.66	0.17	79.77	0.22
		Distance O-Y	120.52	0.25	120.21	0.31

**Table 2:** Percentage differences between the mean distances obtained by the videogrammetry system and Vicon^®^ with respect to the Vicon^®^ values at different distances and marker velocities.

Working Volume Distance	Markers Velocity	Distance O-X	Distance O-Y
Close-range volume	Statics	0.09%	0.04%
	Low velocity	0.11%	0.15%
	Higher velocity	0.01%	0.02%
More distant volume	Statics	0.08%	0.29%
	Low velocity	0.05%	0.10%
	Higher velocity	0.14%	0.26%

**Table 3:** Percentage differences between the mean distances obtained by both systems and the caliper measures with respect to the caliper values.

		Videogrammetry		Vicon^®^	
Working Volume Distance	Markers Velocity	Distance O-X	Distance O-Y	Distance O-X	Distance O-Y
Close-range volume	Statics	0.59%	0.41%	0.49%	0.37%
	Low velocity	0.51%	0.17%	0.63%	0.33%
	Higher velocity	0.55%	0.01%	0.56%	0.02%
More distant volume	Statics	0.82%	0.38%	0.75%	0.09%
	Low velocity	0.63%	0.33%	0.68%	0.23%
	Higher velocity	0.55%	0.21%	0.70%	0.05%

**Table 4:** Computed mean distances and SD (mm) between O-X and O-Y using the videogrammetry system in the three tests and the $rTEM_{intra}$ of each test.

Test	Distance O-X		Distance O-Y	
	Mean (SD)	$rTEM_{intra}$	Mean (SD)	$rTEM_{intra}$
Limited illumination	79.76 (0.13)	0.11%	120.79 (0.04)	0.02%
Variable illumination	79.79 (0.07)	0.06%	120.85 (0.17)	0.10%
Cluttered scenario	79.93 (0.09)	0.08%	120.55 (0.04)	0.02%

## Data Availability

The data presented in this study are available on request from the corresponding author.

## Supplementary Materials

The following supporting information can be downloaded at: https://www.mdpi.com/article/10.3390/s25164900/s1. Code of the videogrammetry system developed in Matlab^®^.
